# Spatial architecture shapes programmed cell death-associated immune niches and therapeutic resistance in clear cell renal cell carcinoma

**DOI:** 10.3389/fonc.2026.1952158

**Published:** 2026-10-12

**Authors:** Yu Yin, Junlong Wang, Dengxiong Li, Dandan Qiu

**Affiliations:** Department of Urology, The First Affiliated Hospital of Zhejiang Chinese Medical University (Zhejiang Provincial Hospital of Chinese Medicine), Hangzhou, China

**Keywords:** clear cell renal cell carcinoma, ferroptosis, immune niche, programmed cell death, renal cell carcinoma, single-cell genomics, spatial multi-omics, therapeutic resistance

## Abstract

Clear cell renal cell carcinoma (ccRCC) is characterized by VHL-HIF pathway alterations, lipid remodeling, abnormal angiogenesis, and marked immune heterogeneity. These features create a highly vascular yet unevenly perfused microenvironment in which vascular dysfunction, hypoxia, stromal barriers, and myeloid clearance influence whether cell death becomes immunologically productive. Although immune checkpoint inhibitor combinations, vascular endothelial growth factor receptor tyrosine kinase inhibitors, and HIF-2α inhibitors have improved outcomes, primary and acquired resistance and discordant responses among lesions remain common. Bulk PD-L1 expression, immune-cell abundance, and cell-death-related transcript signatures do not resolve this heterogeneity. Following current nomenclature, regulated cell death (RCD) is the mechanistically preferred umbrella term; we retain programmed cell death (PCD) in the conceptual label because it remains widely used in the RCC oncology literature, while requiring RCD execution-level evidence for mechanistic claims. Single-cell and spatial multi-omics studies increasingly show that the identity of the dying cell, execution of the death program, accessibility of death-derived material, and the recipient immune cells operate as a coupled local system. We define a PCD-associated immune niche as a spatial tissue state in which representative, non-exclusive immune-routing axes can direct tumor-cell death toward cDC1-mediated cross-presentation and adaptive antitumor immunity, TAM-dominant efferocytosis and tissue repair, or intermediate mixed states. This Review focuses predominantly on ccRCC, evaluates ferroptosis, apoptosis, pyroptosis, necroptosis, and PANoptosis, and briefly considers emerging RCD modalities for which ccRCC evidence is less mature. We further propose composite spatial biomarkers and mechanism-guided combinations that link cellular source, execution evidence, immune routing, and clinical outcome.

## 1 Introduction

Systemic treatment of advanced RCC has shifted from single-agent targeted therapy toward combinations that pair PD-1/PD-L1 blockade with either CTLA-4 blockade or VEGFR tyrosine kinase inhibition, while HIF-2α inhibition has introduced an additional mechanism-based treatment class. CheckMate 214, KEYNOTE-426, CheckMate 9ER, and CLEAR established several first-line standards, and belzutifan confirmed the clinical tractability of the VHL-HIF axis (1–11). Therapeutic diversification, however, has not eliminated biological heterogeneity. Some tumors contain abundant CD8^+^ T cells yet fail to undergo durable regression, whereas others initially respond and later progress at selected sites.

These clinical patterns illustrate why immune-cell abundance cannot be equated with effective antitumor immunity. In RCC, T cells may remain confined to perivascular regions, become trapped within cancer-associated fibroblast (CAF)- and extracellular matrix (ECM)-rich stroma, or enter tumor tissue in terminally exhausted or lipid-peroxidized states. Macrophages can support antigen processing, but they can also remove dying cells through low-inflammatory programs that favor tolerance. Abnormal vessels are likewise more than therapeutic targets: they continually shape hypoxia, acidosis, leukocyte trafficking, and suppressive myeloid recruitment (12–29). Accordingly, PD-L1 expression, CD8^+^ T-cell density, and PCD-associated gene scores have limited explanatory or predictive value when considered in isolation.

A spatially resolved view of tumor-cell death provides a more coherent explanation for these observations. Dying cells may expose or release tumor antigens and damage-associated molecular patterns (DAMPs) that support dendritic-cell maturation and cross-presentation. The same material may instead be engulfed by tumor-associated macrophages (TAMs), with outcomes that range from tolerogenic tissue repair to inflammatory or antigen-presenting states depending on macrophage phenotype and context. Similarly, brief and localized pyroptotic or necroptotic inflammation may assist immune priming, whereas persistent lytic signaling may recruit myeloid-derived suppressor cells (MDSCs) and sustain suppressive inflammation (30–37). The novelty of this Review is therefore not the enumeration of individual death pathways, but a continuous validation framework linking cellular source → death execution → spatial accessibility → recipient cell → immune outcome → treatment response. Unless otherwise specified, mechanistic conclusions in the main text refer primarily to ccRCC; evidence from non-clear-cell RCC or other malignancies is explicitly identified as supportive or hypothesis-generating. Therefore, PCD-associated immune niche is used as a conceptual term, whereas individual death mechanisms are discussed according to current RCD nomenclature.

## 2 PCD-associated immune niches: concept and evidence framework

### 2.1 From individual death pathways to spatial immune outcomes

A PCD-associated immune niche can be defined as a local tissue state in which a verifiable death-execution event occurs within a specified cell population and the resulting antigens, DAMPs, lipid mediators, and inflammatory signals are processed by neighboring immune, stromal, and vascular compartments. Four questions are inseparable within this framework: which cell population is dying; which death program has reached the execution phase; where the event occurs; and which cells capture and process death-derived material. The cDC1-associated and TAM-associated routes used throughout this Review are representative dominant axes rather than mutually exclusive binary outcomes (30–37).

This framework explains why the same nominal death program can produce opposing outcomes. Ferroptosis in malignant cells may cooperate with CD8^+^ T-cell-mediated tumor killing, whereas ferroptotic injury to effector T cells compromises immunotherapy. Spatially confined pyroptosis may increase antigen accessibility, but prolonged inflammatory signaling can reinforce myeloid suppression. Therapy-induced apoptosis may contribute to immunogenic cell death when accompanied by DAMP exposure and type 1 conventional dendritic cell (cDC1)-mediated presentation, yet the same apoptotic material may instead become a substrate for TAM-mediated efferocytosis. The immune significance of PCD therefore cannot be assigned from the pathway name alone (34–44). **Figure 1** summarizes this integrated spatial framework and its principal immune-routing outcomes.

**Figure 1:**
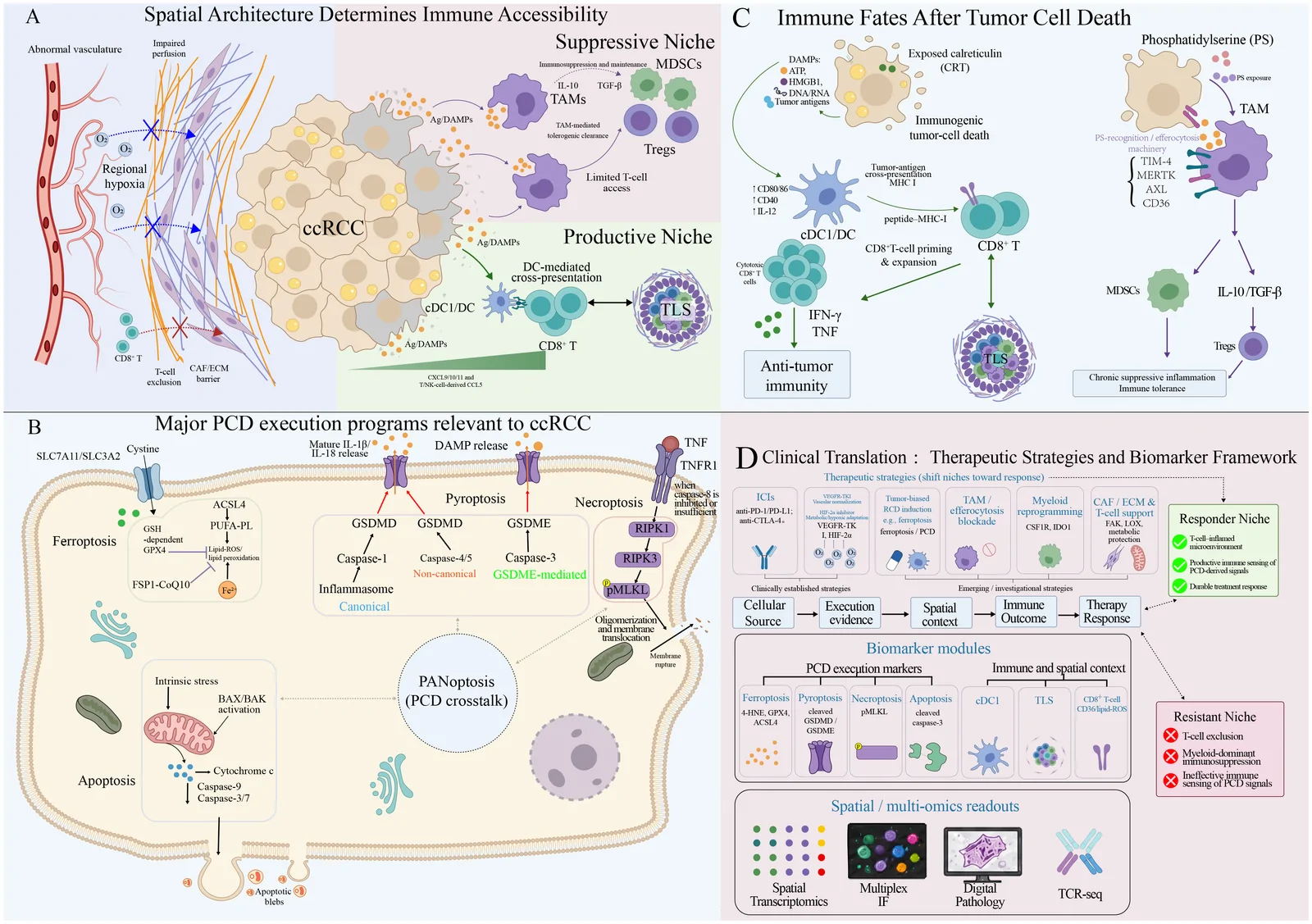
Integrated spatial framework of programmed cell death-associated immune niches in clear cell renal cell carcinoma. **(A)** Abnormal vasculature, regional hypoxia, and cancer-associated fibroblast/extracellular matrix barriers determine the accessibility of tumor-cell death products and separate suppressive from immunologically productive neighborhoods. **(B)** Ferroptosis, apoptosis, pyroptosis, and necroptosis represent distinct execution programs, whereas PANoptosis denotes coordinated crosstalk among pyroptotic, apoptotic, and necroptotic machinery and does not include ferroptosis. **(C)** cDC1-mediated cross-presentation and TAM-mediated efferocytosis are shown as representative dominant immune-routing axes, not mutually exclusive binary outcomes; NK cells, heterogeneous macrophage states, and mixed myeloid neighborhoods can generate intermediate outcomes. **(D)** Clinical translation requires an integrated chain linking cellular source, execution evidence, spatial context, immune outcome, and treatment response. CAF, cancer-associated fibroblast; cDC1, type 1 conventional dendritic cell; ccRCC, clear cell renal cell carcinoma; DAMP, damage-associated molecular pattern; ECM, extracellular matrix; HIF-2α, hypoxia-inducible factor 2 alpha; ICI, immune checkpoint inhibitor; MDSC, myeloid-derived suppressor cell; PCD, programmed cell death; RCC, renal cell carcinoma; TAM, tumor-associated macrophage; TCR-seq, T-cell receptor sequencing; TLS, tertiary lymphoid structure; Treg, regulatory T cell; VEGFR-TKI, vascular endothelial growth factor receptor tyrosine kinase inhibitor.

### 2.2 Evidence hierarchy and interpretive boundaries

The evidence can be organized into three levels. The first comprises direct observations in ccRCC patient cohorts, including single-cell genomics, T-cell receptor sequencing, spatial multi-omics, and studies linking tertiary lymphoid structures (TLS) or defined cellular neighborhoods to treatment outcome. The second includes execution-level experiments in ccRCC cells, organoids, animal models, and tissue specimens. The third consists of mechanisms established in other tumor types or in fundamental immunology. Such transferable evidence is valuable for hypothesis generation, but it should not be presented as an established ccRCC-specific causal pathway without direct validation (17–29, 34–60). Following current cell-death nomenclature, RCD is the mechanistically broader term, whereas PCD more strictly refers to RCD occurring as part of physiological programs. We retain PCD in the conceptual label to preserve continuity with the terminology widely used in RCC transcriptomic and oncology literature; however, all mechanistic assertions are evaluated using execution-level RCD criteria (30). Cross-tumor single-cell and spatial models are therefore treated as methodological analogies rather than RCC-specific biological evidence (61, 62).

A central distinction is that pathway-associated expression does not establish completed cell death. mRNA abundance, gene-set enrichment, and prognostic signatures indicate pathway activity, stress, or susceptibility, but cannot by themselves demonstrate ferroptosis, pyroptosis, necroptosis, or apoptosis. A defensible interpretation requires execution-level evidence: lipid peroxidation together with iron dependence and ferroptosis-selective rescue; GSDMD/GSDME cleavage with pore-dependent membrane permeability; MLKL phosphorylation, oligomerization, and membrane localization; or caspase/PARP cleavage with compatible morphology and pathway intervention. **Table 1** summarizes the minimum evidence needed to define a PCD-associated immune niche (30, 63–67).

**Table 1:** Operational framework for defining a programmed cell death-associated immune niche in clear cell renal cell carcinoma.

Domain	Question to be resolved	Evidence required for a defensible interpretation	What the data do not establish on their own	Relevance to RCC biology and clinical translation
Cellular provenance	Which compartment is dying—malignant epithelial cells, CD8^+^ T cells, NK cells, TAMs, endothelial cells, or another stromal population?	Co-detection of a lineage marker with an execution marker by multiplex IF/IHC, imaging mass cytometry, or spatial proteomics; single-cell transcriptomic or proteomic evidence should be supported by tissue localization whenever possible.	Bulk RNA abundance, a regional expression score, or an unsegmented tissue stain cannot identify the cellular source of death.	The direction of the immune effect depends on the dying cell. Tumor-cell death may support tumor control, whereas death of CD8^+^ T or NK cells removes effector function and may favor resistance.
Execution of the death program	Has the pathway reached the execution phase, or is the tissue merely primed or stressed?	At least one execution-level readout plus orthogonal confirmation: lipid peroxidation with ferroptosis rescue; GSDMD/GSDME cleavage and pore-dependent permeability; pMLKL membrane localization; caspase/PARP cleavage with compatible morphology.	mRNA expression, gene-set enrichment, or a prognostic signature cannot by itself demonstrate ferroptosis, pyroptosis, necroptosis, or apoptosis.	Execution-level evidence identifies the biologically active node and avoids attributing a treatment response to a pathway that is transcriptionally enriched but not functionally engaged.
Spatial accessibility	Can antigens and danger signals released from dying cells reach cDC1/DCs, and TLS-associated antigen-presentation regions, thereby enabling subsequent CD8^+^ T-cell priming, or are they separated by vessels, hypoxia, and stroma?	Quantitative neighborhood analysis; distance from PCD-positive tumor cells to cDC1, CD8^+^ T cells, TAMs, and TLS; assessment of abnormal vasculature, hypoxia, CAF density, collagen organization, and tumor-border exclusion.	Cell counts alone do not show whether immune cells contact the tumor, remain trapped in perivascular/stromal zones, or are absent from the region in which death occurs.	Spatial accessibility helps explain discordant responses between lesions and within a single tumor, even when overall immune-cell abundance is similar.
Immune routing of dying-cell material	Which recipient populations dominate, and are intermediate or mixed routing states present rather than a strictly cDC1-versus-TAM binary outcome?	Assess cDC1/DC uptake and cross-presentation, NK- and CD8^+^ T-cell proximity/function, macrophage-state markers, MERTK/AXL/TIM-4/CD36-associated engulfment, inflammatory mediators, and direct uptake events within the same spatial region.	The presence of tumor-cell death, DAMP release, cDC1, or TAM markers alone does not establish a single immune fate. M1/M2 labels and receptor expression are insufficient to define macrophage function without spatial and functional evidence.	The framework should treat cDC1-dominant cross-presentation and TAM-dominant efferocytosis as representative poles within a continuum that also includes NK-cell effects, mixed macrophage states, and other myeloid populations.
Temporal and treatment context	At what stage of disease and treatment is the niche being measured, and is the signal transient or sustained?	Paired pretreatment, on-treatment, and progression samples; serial blood or imaging correlates where tissue is unavailable; documentation of treatment exposure, sampling interval, and inflammatory duration.	A single pretreatment biopsy cannot determine whether a niche is an intrinsic cause of resistance, a treatment-induced adaptation, or a consequence of progression.	Longitudinal assessment is required to distinguish baseline resistance from therapy-driven selection and to define when a PCD-directed combination is most likely to be effective.

A mechanistic claim is strongest when cellular provenance, execution, spatial accessibility, recipient-cell state, immune routing, and temporal context are measured in the same specimen or in a prospectively linked specimen set. The productive-versus-tolerogenic distinction is a useful organizing axis, not a binary classification of all in vivo immune outcomes.

CAF, cancer-associated fibroblast; cDC1, type 1 conventional dendritic cell; CRT, calreticulin; DC, dendritic cell; DAMP, damage-associated molecular pattern; IF, immunofluorescence; IHC, immunohistochemistry; NK, natural killer; PCD, programmed cell death; RCC, renal cell carcinoma; TAM, tumor-associated macrophage; TLS, tertiary lymphoid structure.

### 2.3 Literature search and evidence selection

This article is a narrative, mechanism-oriented review rather than a systematic review or meta-analysis. PubMed/MEDLINE was used as the primary bibliographic database and was updated through 18 August 2026. Searches combined “renal cell carcinoma” or “clear cell renal cell carcinoma” with terms related to ferroptosis, apoptosis, pyroptosis, necroptosis, PANoptosis, cuproptosis, disulfidptosis, parthanatos, lysosome-dependent cell death, entosis, immune checkpoint therapy, dendritic cells, macrophages, natural killer cells, tertiary lymphoid structures, single-cell sequencing, spatial transcriptomics, spatial proteomics, radiomics, and treatment resistance. Reference lists of key primary studies and reviews were also screened, and ClinicalTrials.gov was consulted when discussing investigational therapeutic agents. Evidence was prioritized in the following order: direct ccRCC patient-tissue observations; functional or execution-level evidence in ccRCC models; non-clear-cell RCC evidence; and cross-tumor or fundamental mechanistic evidence. Cross-tumor studies were retained only when they clarified a conserved mechanism or a transferable analytical approach and were explicitly described as analogical rather than ccRCC-specific. Because the review is narrative, no pooled effect estimate or PRISMA-style claim of exhaustive study capture is made.

## 3 ccRCC-specific biological foundations: metabolism, vasculature, and immunity

### 3.1 VHL-HIF dysregulation creates an abnormal vascular ecology

Loss of VHL function and persistent HIF signaling are defining molecular features of ccRCC. HIF activation upregulates VEGF, glucose transport, and hypoxia-adaptation programs, generating tumors that are richly vascularized but structurally disorganized and unevenly perfused. These vessels regulate far more than nutrient delivery: they shape oxygenation, extracellular acidity, leukocyte adhesion and extravasation, and recruitment of suppressive myeloid populations. VEGFR-TKIs may therefore remodel PCD-associated niches not only through antiangiogenic activity, but also through dose- and time-dependent vascular normalization, relief of hypoxia, and changes in the myeloid compartment (12–17, 68, 69).

### 3.2 Lipid accumulation creates dependence on ferroptosis-suppressive systems

The clear-cell morphology of ccRCC is largely associated with extensive lipid and glycogen accumulation. A large pool of oxidizable polyunsaturated phospholipids, persistent redox stress, and dependence on glutathione homeostasis increase reliance on the SLC7A11-GSH-GPX4 and FSP1-CoQ10 defense systems. When these defenses fail, ACSL4-dependent remodeling of polyunsaturated fatty acid-containing phospholipids and redox-active Fe^2+^ drive the propagation of membrane lipid peroxidation (45, 46, 63–67, 70–74). The metabolic program that supports clear-cell survival therefore also creates a potentially actionable vulnerability (45, 46). Recent studies have further demonstrated that lipid metabolic remodeling and lipid-associated regulatory mechanisms contribute to ccRCC progression and adaptation to the tumor microenvironment, highlighting the importance of metabolic regulation in determining tumor-cell vulnerability and therapeutic response (75, 76). RCC-specific studies of ferroptosis resistance and sorafenib-associated ferroptosis further reinforce the importance of redox defense when interpreting therapeutic vulnerability (77, 78).

### 3.3 Immune-rich does not necessarily mean immune-effective

ccRCC frequently contains abundant T-cell and myeloid infiltrates, yet high immune-cell density does not consistently predict a favorable outcome. Single-cell studies reveal progressive T-cell dysfunction, myeloid reprogramming, abnormal endothelial states, and altered antigen-presentation programs in malignant cells during disease evolution (17–25). The conventional “hot” versus “cold” classification is therefore inadequate. A single lesion may simultaneously contain immune-excluded borders, suppressive myeloid neighborhoods, TLS-associated immune-active regions, hypoxic or necrotic margins, and highly vascular territories. A PCD event occurring in each of these spatial units is likely to have a different immune consequence (26–29).

## 4 Spatial architecture controls the immune accessibility of PCD-derived signals

### 4.1 Abnormal vasculature and hypoxia as upstream spatial gates

VEGF-driven tumor vessels typically show irregular lumina, heterogeneous perfusion, excessive permeability, and endothelial dysfunction. Despite their numerical abundance, these vessels often fail to provide stable and effective tissue perfusion, producing regions of hypoxia, acidosis, and uneven nutrient supply. Vascular and endothelial abnormalities also impair lymphocyte adhesion, transendothelial migration, and intratumoral penetration while favoring TAM-, MDSC-, and regulatory T-cell-associated suppression. When cell death occurs in a poorly perfused tumor core, even substantial release of DAMPs and tumor antigens may fail to generate adaptive immunity because cDC1 are scarce, effector lymphocytes cannot reach the site, or sustained hypoxia compromises antigen presentation and immune function. Dying-cell material may instead be captured preferentially by nearby myeloid cells and incorporated into tissue-repair or tolerogenic programs. Abnormal vasculature is therefore an important upstream spatial gate that determines whether PCD-derived signals can be accessed, recognized, and used by the immune system (12–17, 25, 69).

### 4.2 CAF/ECM barriers separate immune infiltration from productive contact

CAFs continuously remodel the ECM through the production of collagen, fibronectin, TGF-β, and multiple chemokines, creating a stromal barrier with both physical and immunoregulatory functions. ccRCC specimens may contain substantial numbers of CD8^+^ T cells that remain confined to perivascular or CAF/ECM-rich stromal regions and fail to enter malignant-cell nests, thereby producing an immune-excluded phenotype. In a PCD-associated niche, this spatial decoupling not only limits direct contact between effector T cells and malignant cells, but can also impede the movement of tumor antigens and DAMPs toward cDC1 and TLS, reducing antigen uptake, cross-presentation, and local adaptive immune priming. TAMs located closer to the dying cells may then capture the material first and route it toward low-inflammatory tissue repair and immune tolerance. Assessment of PCD-associated immunity should therefore quantify distance, neighborhood relationships, and direct contacts among dying cells, cDC1, CD8^+^ T cells, TAMs, and TLS rather than relying on cell counts alone (17–29, 80, 81).

### 4.3 Suppressive myeloid regions and TLS-associated regions process death signals differently

Cellular neighborhoods enriched in TAMs, MDSCs, and regulatory T cells are more likely to route dying-cell material toward tolerogenic clearance, tissue repair, and chronic suppressive inflammation. By contrast, regions containing mature cDC1, CD8^+^ T cells with recoverable effector potential, and well-organized TLS are better positioned to support antigen uptake, cross-presentation, local T-cell activation, clonal expansion, and immune memory (18–29). RCC studies further suggest that TLS maturity and tissue-resident exhausted T-cell states jointly associate with response to PD-1 blockade. These observations imply that spatial organization, cellular functional state, and intercellular relationships may be more informative determinants of therapeutic benefit than the abundance of any single immune population (27–29, 34, 37, 82, 83).

Future spatial studies should register tissue morphology, cellular neighborhoods, and execution-level PCD evidence within the same coordinate system. In regions enriched for 4-hydroxynonenal or oxidized phospholipids, for example, GPX4 and ACSL4 should be assessed together with the spatial distribution of cDC1, CD8^+^ T cells, and TLS. Sites positive for cleaved GSDMD/GSDME or phosphorylated MLKL should be evaluated for IL-1-family signaling, myeloid composition, and local inflammatory state. Around cleaved-caspase-3-positive malignant cells, TAM expression of MERTK, AXL, and TIM-4 should be examined alongside IL-10 and TGF-β. These markers should not be interpreted in isolation; cell-of-origin assignment, spatial proximity, and functional state must corroborate one another. Only a continuous chain linking the dying-cell source, execution evidence, local neighborhood, recipient cell, and immune outcome can establish whether a death event creates an immunologically productive niche or is absorbed into a suppressive clearance network (30–58, 63–67, 70–74, 82, 83).

## 5 Major regulated cell-death programs in ccRCC and their immune outputs

### 5.1 Ferroptosis: a bidirectional axis linking tumor vulnerability and T-cell fitness

Ferroptosis is an iron-dependent RCD driven by phospholipid peroxidation. In ccRCC, the most relevant execution balance involves cystine uptake through system xCT (SLC7A11/SLC3A2), GSH-dependent GPX4 activity, the parallel FSP1-CoQ10 defense system, ACSL4-dependent formation of oxidizable phospholipids, and redox-active iron (63–67, 70–74). Because ccRCC is lipid-rich and strongly dependent on glutathione-centered redox control, this network serves both as a survival requirement and as a potential therapeutic vulnerability (45, 46).

Antitumor immunity can also lower the ferroptosis threshold of malignant cells. IFN-γ released by activated CD8^+^ T cells suppresses tumor-cell SLC3A2 and SLC7A11, limiting cystine uptake and GSH synthesis and thereby weakening GPX4-dependent protection (38). ACSL4-dependent phospholipid remodeling supplies the oxidizable membrane substrates required for this immune-driven process (39). The same lipid-rich environment can injure CD8^+^ T cells through CD36-dependent lipid uptake and peroxidation (40). Evidence in ccRCC is strongest for tumor-cell redox dependence and functional ferroptosis susceptibility, including GPX4/SLC7A11-linked resistance phenotypes and sorafenib-associated ferroptotic death in RCC models (60). The major unresolved issue is cell-type selectivity: prospective patient-tissue studies must show that a candidate intervention increases execution in malignant cells without simultaneously impairing effector lymphocytes.

### 5.2 Apoptosis is not intrinsically immunogenic

Apoptosis is executed through mitochondrial BAX/BAK-caspase signaling or extrinsic death-receptor/granzyme pathways and generally preserves plasma-membrane integrity during its early phase. It is therefore not intrinsically immunogenic. Surface calreticulin exposure, extracellular ATP/HMGB1, and effective cDC1-mediated antigen uptake can convert selected treatment-induced apoptotic events into immunogenic cell death, whereas rapid macrophage efferocytosis can favor tissue repair and tolerance (30–33). In ccRCC, apoptosis itself is well established, but the spatial routing of apoptotic material toward productive cross-presentation versus efferocytosis remains insufficiently resolved in patient tissue.

### 5.3 Pyroptosis lies between immune activation and chronic suppression

Pyroptosis is gasdermin-mediated lytic RCD. Canonical inflammasome-caspase-1 signaling and human non-canonical caspase-4/5 signaling can cleave GSDMD, whereas caspase-3 cleavage of GSDME can convert selected apoptotic stimuli into pore-forming death (41, 42, 47–51). Gasdermin pores permit DAMP release, but mature IL-1β/IL-18 still require appropriate cytokine-processing machinery. Evidence in ccRCC remains limited: most human data are transcriptional or associative, and direct spatial demonstration of cleaved gasdermins, pore-forming fragments, cellular source, and treatment response is still sparse (59).

Accordingly, pyroptosis should not be inferred from IL-1β abundance or a transcript score alone; cleaved GSDMD/GSDME, pore-forming fragments, membrane-permeability phenotypes, and cell-type assignment are required before linking pyroptosis to spatial immune routing in ccRCC (37, 41, 42, 47–51).

### 5.4 Necroptosis is molecularly defined regulated membrane rupture

Necroptosis is defined by RIPK3-dependent MLKL phosphorylation, oligomerization, and membrane translocation, often downstream of TNF/TNFR signaling when caspase-8 activity is constrained (43, 44, 52–55). Histological necrosis or nonspecific membrane rupture is not sufficient evidence. In ccRCC, necroptosis-related signatures and pathway crosstalk have been reported, but execution-level pMLKL evidence with cell-type and spatial assignment remains limited; the confidence level is therefore lower than for ferroptosis or apoptosis (59).

### 5.5 PANoptosis is an integrative platform that remains to be established in ccRCC

PANoptosis denotes stimulus-dependent integration of pyroptotic, apoptotic, and necroptotic machineries through multiprotein PANoptosome complexes (56–58). Ferroptosis is not a component of PANoptosis, and co-expression of multiple pathway genes cannot establish PANoptosis. In ccRCC, direct evidence remains hypothesis-generating; future studies should demonstrate concurrent execution of multiple arms, physical complex formation, and coordinated attenuation after perturbation of a shared upstream regulator (59). **Table 2** summarizes the defining machinery, verification strategy, immune consequences, and interpretive boundaries of the major PCD programs.

**Table 2:** Major regulated cell-death programs relevant to ccRCC: execution criteria, immune consequences, evidence hierarchy, confidence, and unresolved questions.

PCD program	Defining execution machinery	Preferred verification strategy	Immune consequences most relevant to RCC	Evidence hierarchy, confidence, and key unresolved question
Ferroptosis	SLC7A11–GSH–GPX4 and FSP1–CoQ10 suppress membrane lipid peroxidation; ACSL4-dependent PUFA-phospholipid remodeling and redox-active Fe²^+^ increase susceptibility.	Demonstrate lipid peroxidation with C11-BODIPY, oxidized phospholipids, 4-HNE, or MDA; confirm iron and GPX4 dependence; show rescue with ferrostatin-1, liproxstatin-1, or another selective intervention. Tissue studies should co-localize these readouts with tumor or immune-cell markers.	Tumor-cell ferroptosis can increase antigen and oxidized-lipid release and may cooperate with IFN-γ-producing CD8^+^ T cells. In contrast, CD36-driven lipid uptake and peroxidation in intratumoral CD8^+^ T cells reduce cytokine production and cytotoxicity.	Patient tissue: ccRCC redox dependence and ferroptosis-related states are supported by tissue and single-cell data. Functional ccRCC: strong evidence for GPX4/SLC7A11/NRF2-linked susceptibility and resistance phenotypes, including sorafenib-associated ferroptosis (45, 46, 60, 77, 78). Cross-tumor support: CD8^+^ T-cell control of tumor ferroptosis and CD36-dependent T-cell injury (38–40). Confidence: moderate-to-high for tumor vulnerability; lower for spatial immune routing. Key gap: tumor-selective induction with prospective execution markers.
Apoptosis and apoptosis-associated immunogenic cell death	Mitochondrial BAX/BAK activation, cytochrome-c release, caspase-9, and caspase-3/7; extrinsic death-receptor and granzyme pathways may converge on the same execution phase.	Use cleaved caspase-3 and PARP with cytochrome-c release or mitochondrial permeabilization, compatible morphology, and pathway inhibition. To support immunogenic apoptosis, additionally document surface CRT, extracellular ATP/HMGB1, DC uptake, or cross-presentation.	Apoptosis supplies tumor antigens but is commonly removed without overt inflammation. It becomes immunologically productive only when danger signals and competent antigen presentation accompany cell clearance.	Patient tissue: apoptosis is readily demonstrable, but immunogenic routing is less well resolved. Functional RCC: apoptosis is established; evidence that apoptotic material is converted into durable cDC1-dependent immunity in ccRCC is limited. Cross-tumor/fundamental support: strong for CRT/ATP/HMGB1-associated immunogenic cell death and efferocytosis (30–37). Confidence: high for apoptosis, moderate for immune routing. Key gap: spatially link apoptotic tumor cells to cDC1/TLS versus macrophage uptake.
Pyroptosis	Canonical inflammasome–caspase-1–GSDMD; non-canonical caspase-4/5/11–GSDMD; caspase-3–GSDME can convert selected apoptotic stimuli into pore-forming death.	Detect the pore-forming N-terminal fragment of GSDMD or GSDME, membrane permeability or pore formation, cell swelling, and release of mature IL-1β/IL-18 where applicable. Genetic or pharmacological interruption of the relevant caspase–gasdermin axis should reduce the phenotype.	Spatially confined tumor-cell pyroptosis may enhance antigen release, DC activation, and T-cell recruitment. Sustained IL-1-family signaling can instead recruit suppressive myeloid cells, activate CAFs, and reinforce abnormal angiogenesis.	Patient tissue: predominantly transcriptomic/associative in ccRCC. Functional RCC: execution-level evidence is limited. Cross-tumor support: strong for GSDMD/GSDME execution and immune activation (41, 42, 47–51). Confidence: low-to-moderate in ccRCC. Key gap: cleaved gasdermins, cellular source, pore formation, and treatment-response linkage in patient tissue.
Necroptosis	TNF/TNFR signaling through RIPK1 and RIPK3, followed by MLKL phosphorylation, oligomerization, and plasma-membrane disruption; other upstream stimuli may converge on RIPK3–MLKL.	Show pMLKL with membrane localization or oligomerization, RIPK3 activation, loss of membrane integrity, and reduction of death after pathway-specific genetic or pharmacological inhibition.	Release of nucleic acids, DAMPs, and cytosolic proteins can support cross-priming, but persistent lytic inflammation may also maintain myeloid recruitment and tissue-repair programs.	Patient tissue: histological necrosis is common but is not evidence of necroptosis. Functional RCC: pathway signatures/crosstalk are reported, but pMLKL-based spatial execution evidence remains sparse (43, 44, 52–55, 59). Cross-tumor support: strong for RIPK3–MLKL execution. Confidence: low-to-moderate in ccRCC. Key gap: cell-type-resolved pMLKL oligomerization/membrane localization with functional rescue.
PANoptosis	Coordinated engagement of pyroptotic, apoptotic, and necroptotic machineries through a stimulus-dependent multiprotein platform often referred to as a PANoptosome.	Demonstrate concurrent activation of at least two or three execution arms, evidence of complex formation or physical interaction, and coordinated loss of the composite phenotype after perturbing a shared upstream regulator.	The resulting signal is more complex than any single PCD program and may combine cytokine release, membrane lysis, antigen availability, and compensatory switching between death pathways.	Patient tissue: no convincing direct demonstration in ccRCC. Functional RCC: currently hypothesis-generating. Cross-tumor support: established concept of integrated pyroptotic, apoptotic, and necroptotic machinery (56–58). Confidence: low in ccRCC. Key gap: PANoptosome assembly plus concurrent execution of multiple arms and coordinated pathway perturbation (59).
Emerging RCD modalities	Cuproptosis involves copper-dependent stress on lipoylated mitochondrial proteins; disulfidptosis involves disulfide stress and actin-cytoskeleton collapse. Parthanatos, lysosome-dependent cell death, and entotic cell death represent additional mechanistically defined RCD categories.	Use modality-specific execution criteria rather than transcript signatures alone. For cuproptosis, demonstrate copper dependence and FDX1/lipoylated-protein pathway engagement; for disulfidptosis, demonstrate disulfide stress with actin-cytoskeleton collapse. Apply accepted mechanistic criteria for parthanatos, lysosome-dependent cell death, or entotic cell death.	The immune consequences of these modalities in ccRCC remain incompletely characterized and should not be assumed to mirror ferroptosis, pyroptosis, or necroptosis.	ccRCC evidence: functional evidence supports FDX1-linked cuproptosis and sunitinib resistance (84), whereas disulfidptosis evidence remains predominantly transcriptomic/prognostic (85). For parthanatos, lysosome-dependent cell death, and entotic cell death, direct cell-type-resolved and spatial ccRCC evidence is currently insufficient; the broader mechanistic framework is supported by RCD literature (84). Confidence: low-to-moderate for cuproptosis and low for the other emerging modalities.

The hierarchy distinguishes direct patient-tissue evidence, ccRCC functional evidence, and cross-tumor/fundamental evidence. No single marker or transcript signature is sufficient to establish an execution program.

4-HNE, 4-hydroxynonenal; CAF, cancer-associated fibroblast; CRT, calreticulin; GSDMD/E, gasdermin D/E; GSH, glutathione; GPX4, glutathione peroxidase 4; MDA, malondialdehyde; MLKL, mixed-lineage kinase domain-like protein; PARP, poly(ADP-ribose) polymerase; PUFA, polyunsaturated fatty acid; RCC, renal cell carcinoma; RIPK, receptor-interacting protein kinase; RCD, regulated cell death.

### 5.6 Emerging RCD modalities beyond the core framework

Cuproptosis is triggered by copper-dependent stress on lipoylated mitochondrial proteins; in ccRCC, FDX1-linked cuproptosis has experimental support and has been connected to sunitinib resistance, although patient-level spatial execution markers are not yet established (84). Disulfidptosis results from disulfide stress and actin-cytoskeleton collapse in SLC7A11-high cells; ccRCC studies are currently dominated by transcriptomic/prognostic modeling rather than execution-level validation (85). Parthanatos, lysosome-dependent cell death, and entotic cell death are recognized mechanistic categories within the broader RCD literature, but convincing cell-type-resolved, execution-level, and spatial evidence in ccRCC remains insufficient (30). We therefore discuss these modalities explicitly but do not place them on the same evidentiary tier as the core pathways in the present niche framework.

## 6 Immune fates after tumor-cell death

### 6.1 Productive immune sensing: DAMPs, cross-presentation, and adaptive priming

Tumor cells undergoing immunogenic death can expose CRT and release extracellular ATP, HMGB1, tumor antigens, and nucleic acids. In a permissive spatial context, cDC1 can capture dying-cell antigens and support MHC-I cross-presentation, costimulation, and expansion of tumor-reactive CD8^+^ T cells (31–33). Mature TLS may further organize local B/T-cell interactions and clonal expansion (27–29, 82, 83). This cDC1/TLS route is best viewed as one representative productive axis rather than the only possible immune fate of dying-cell material.

### 6.2 Efferocytosis routes dying-cell material toward tolerance

Apoptotic tumor cells expose phosphatidylserine (PS) and can be engulfed through efferocytosis-associated receptors. MERTK, AXL, and TYRO3 often recognize PS through bridging ligands such as Gas6 or protein S, whereas TIM-4 and CD36 participate in binding and uptake. Sustained efferocytosis can support IL-10, TGF-β, VEGF, and tissue-repair programs (34, 37). Phagocytic checkpoints such as LILRB1-MHC-I and CD24-Siglec-10 act at a distinct stage by regulating whether target cells are engulfed (35, 36). These mechanisms should therefore not be collapsed into a single ‘tolerogenic clearance’ pathway: their net effect depends on macrophage state, ligand availability, coexisting inflammatory cues, and whether antigen presentation is preserved.

### 6.3 Immune routing is non-binary: NK cells and macrophage-state heterogeneity

NK cells provide an additional spatial effector layer through perforin/granzyme release, TRAIL signaling, and cytokine production, but RCC studies indicate that intratumoral NK-cell cytotoxicity can be impaired through changes in activating receptors such as DNAM-1 and NKp30/NCR3 (86, 87). Their proximity to tumor cells, vascular access, and functional state should therefore be incorporated alongside CD8^+^ T cells when defining productive death niches. Macrophage biology is likewise more complex than a binary M1/M2 classification. Although M1- and M2-like terminology can serve as historical shorthand, single-cell and spatial data in ccRCC support continuous and mixed TAM states with variable inflammatory, angiogenic, lipid-handling, antigen-presenting, and efferocytic programs (17–29, 79). Accordingly, cDC1-dominant cross-presentation and TAM-dominant efferocytosis are presented as representative poles within a broader continuum of immune-routing states.

### 6.4 The duration and location of inflammation determine its direction

Increased DAMP or inflammatory-cytokine levels do not necessarily indicate stronger antitumor immunity. The immune effect depends on signal duration, spatial extent, cellular source, and the identity of the local recipient cells. Brief, localized inflammation that is effectively coupled to cDC1 or mature TLS is more likely to support dying-cell antigen uptake, dendritic-cell maturation, cross-presentation, and adaptive priming. By contrast, sustained and diffuse inflammation concentrated at hypoxic necrotic margins or within TAM/MDSC-rich regions may chronically activate IL-1-family and CXCL/CCL chemokine networks, recruit suppressive myeloid populations, promote aberrant angiogenesis and stromal remodeling, and convert acute inflammation into a tissue-repair-dominated suppressive state. Interpretation of inflammatory PCD therefore requires more than measurement of signal intensity; it must account for timing, spatial distribution, the cellular source of death, local immune composition, and which cells ultimately receive and process the signals (31–33, 37, 41–44, 47–55, 69).

### 6.5 PCD of effector immune cells is itself a treatment constraint

The lipid-rich ccRCC microenvironment can damage tumor-infiltrating CD8^+^ T cells through CD36-mediated uptake of lipids and oxidized lipids, accumulation of reactive oxygen species, and membrane lipid peroxidation, thereby reducing cytotoxicity and cytokine production and increasing susceptibility to ferroptosis-associated injury (40). Persistent antigen stimulation, nutrient competition, hypoxia, and other metabolic stresses can further drive terminal exhaustion, restrict proliferative recovery, and ultimately deplete effector-cell populations. An effective PCD-directed strategy should therefore selectively increase malignant-cell susceptibility to a defined death program while preserving the survival, metabolic fitness, and sustained function of antitumor immune cells. Bulk increases in tissue lipid peroxidation, reactive oxygen species, or inflammatory signaling are not intrinsically favorable pharmacodynamic endpoints. Their therapeutic relevance can be inferred only when the changes are localized to malignant cells and accompanied by preserved effector-cell function and enhanced adaptive immunity (17, 21).

## 7 PCD-associated immune niches and therapeutic resistance in ccRCC

Although immune checkpoint inhibitors (ICIs), VEGFR-TKIs, HIF-2α inhibitors, and local therapies have defined direct targets, their biological effects extend well beyond a single molecular pathway. At the tissue level, each can simultaneously alter perfusion, oxygenation, metabolism, PCD execution, and tumor-immune interactions. A response-permissive niche may require several conditions to converge: CD8^+^ T cells must enter malignant-cell nests and establish productive contact; tumor cells must generate death outputs that can be recognized and used by the immune system; cDC1 and mature TLS must be able to capture, present, and organize responses to death-derived antigens; and tolerogenic myeloid clearance must remain constrained. A resistant niche may instead maintain SLC7A11-GPX4 or FSP1-dependent ferroptosis protection, reduce antigen processing and presentation, strengthen efferocytosis and phagocytic checkpoints, expand CAF/ECM barriers, or exploit lipid and oxidative stress to selectively impair effector T cells (12–40, 45, 46, 68, 69, 80–83). Broader cancer literature likewise indicates that tumor-educated stromal and immune cells and metabolic competition can jointly contribute to checkpoint-blockade resistance; these observations support the framework but remain cross-tumor evidence rather than RCC-specific validation (88, 89).

This model helps explain three recurring clinical observations. First, PD-L1 expression and CD8^+^ T-cell density are imperfect predictors because neither establishes that T cells have entered malignant-cell nests, retained cytotoxic function, or productively used antigens released from dying cells. Second, the activity of ICI-VEGFR-TKI combinations is unlikely to reflect a simple addition of “immune activation” and “antiangiogenesis.” Appropriate vascular normalization, improved local perfusion and oxygenation, and remodeling of suppressive myeloid populations may together increase the spatial accessibility of dying cells, tumor antigens, and DAMPs, thereby improving conversion of PCD-derived signals into adaptive immunity. Third, HIF-2α inhibition may influence not only tumor proliferation but also angiogenesis, metabolic and redox dependence, and the execution thresholds of several PCD programs. Its biological effects should therefore be evaluated using vascular, metabolic, death-execution, and immune-niche readouts in addition to changes in tumor volume (1–17, 21, 26, 68, 69).

PCD-associated immune niches are dynamic rather than fixed. Under sustained treatment pressure, tumor and stromal compartments continue to evolve. Pretreatment, early on-treatment, and progression samples represent the baseline niche, therapy-induced remodeling, and selection of resistant clones and microenvironments, respectively. Resistance studies should therefore not rely exclusively on a single pretreatment biopsy. Longitudinal integration of single-cell genomics, spatial transcriptomics and proteomics, digital pathology, and PCD execution markers can trace changes in malignant-cell state, death programs, and immune neighborhoods within a common temporal and spatial framework. This approach is needed to distinguish early mechanisms that drive resistance from secondary changes that arise after treatment failure (21, 26, 29).

## 8 Clinical translation: composite biomarkers and mechanism-guided combinations

### 8.1 Moving from isolated biomarkers to composite spatial assessment

A clinically informative biomarker system should capture malignant-cell death execution, effector-cell fitness, cDC1 competence, TAM state and efferocytosis, inflammatory output, and tissue organization. Once spatial discovery signals are validated, they may be compressed into multiplex immunofluorescence, RNAscope, digital pathology, or focused targeted panels (17–60, 82, 83) Cross-cancer imaging and machine-learning studies illustrate technical approaches to multimodal prediction, but they are methodological analogies, not biological evidence for RCC PCD niches and require calibration, external validation, and mechanistic interpretability (90, 91). Non-invasive imaging may provide a complementary, although indirect, readout. In ccRCC, CT radiomic signatures have been associated with CD8^+^ T-cell infiltration and PD-L1 expression, radiogenomic models have captured hypoxia-related biomarkers, and radiomic features have shown associations with response-related outcomes during immune checkpoint blockade (92–94). These approaches should be viewed as surrogates that require prospective calibration against spatial tissue measurements rather than replacements for execution-level evidence.

### 8.2 Combination therapy should target the dominant niche bottleneck

Combination therapy should be designed around the dominant limiting step within a given niche rather than by simply adding drug mechanisms. When effector cells have entered the tumor but malignant cells remain highly dependent on SLC7A11-GPX4 or FSP1-mediated ferroptosis defense, a tumor-selective ferroptosis-sensitizing strategy is a testable hypothesis, but cell-type selectivity is essential because lipid peroxidation can simultaneously impair CD8^+^ T-cell function (38–40, 45, 46, 77, 78). When efferocytosis-active macrophages dominate, death induction could be paired experimentally with selective modulation of AXL/MERTK/TIM-4 or macrophage reprogramming. When vascular or CAF/ECM barriers dominate, vascular normalization or matrix-directed intervention may be required before immunogenic death can be productively sensed.

The translational maturity of these strategies is heterogeneous. ICI-VEGFR-TKI combinations and HIF-2α inhibition have established clinical activity in ccRCC, but their benefit has not been prospectively selected using a PCD-niche biomarker. Ferroptosis-directed treatment remains experimental: sorafenib and resistance regulators such as DPP9/NRF2/SLC7A11 provide RCC-specific mechanistic evidence, but no clinically validated, biomarker-selected ferroptosis-induction strategy currently exists (77, 78). For the myeloid axis, selective AXL inhibition with bemcentinib or an anti-AXL antibody has preclinical RCC support (95), whereas the anti-CSF1R antibody cabiralizumab has entered an early-phase combination study that included advanced RCC (NCT03502330). Cabozantinib also inhibits AXL among several kinases, but its established RCC efficacy should not be interpreted as proof of efferocytosis-directed therapy. MERTK- or TIM-4-directed strategies remain investigational in RCC. **Table 3** now labels these differences explicitly as established, early clinical, preclinical, or conceptual (12–42, 44–58, 69, 80–83).

**Table 3:** PCD-associated immune-niche archetypes in ccRCC: identification, therapeutic hypotheses, safety constraints, and translational maturity.

Niche archetype	Biological phenotype and limiting step	Biomarker and spatial criteria for identification	Therapeutic rationale to be tested	Major interpretive or safety concern	Evidence boundary / translational maturity
CD8-proximal, ferroptosis-resistant tumor niche	CD8^+^ T cells are present near malignant cells, but tumor-cell SLC7A11/GPX4/FSP1 activity remains high and lipid peroxidation is low. The bottleneck is resistance at the tumor-cell execution step rather than lack of immune access.	Co-localize tumor markers with SLC7A11, GPX4, FSP1, ACSL4, 4-HNE or oxidized phospholipids; quantify tumor–CD8 contact. In parallel, assess CD36 and lipid ROS specifically in CD8^+^ T cells.	Evaluate ICIs with a tumor-selective ferroptosis-sensitizing strategy in preclinical or biomarker-enriched settings, using dosing or delivery that increases tumor lipid peroxidation without compromising effector-cell viability.	A non-selective increase in lipid oxidation may worsen CD8^+^ T-cell dysfunction. Bulk 4-HNE or MDA cannot distinguish beneficial tumor-cell ferroptosis from harmful immune-cell injury.	Translational maturity: preclinical/experimental. ccRCC redox vulnerability and sorafenib-linked ferroptosis/resistance mechanisms have functional support (45, 46, 75, 76), but no biomarker-selected clinical ferroptosis-induction strategy is validated. Cell-type selectivity remains the principal safety requirement.
TAM–efferocytosis-dominant niche	Apoptotic tumor material is rapidly engulfed by TAMs through MERTK/AXL/TIM-4/CD36-associated programs. Antigen availability does not translate into cDC1 activation, and IL-10/TGF-β-rich tissue repair predominates.	Measure cleaved caspase-3 in tumor cells together with TAM receptor expression, direct engulfment events, phosphatidylserine exposure, IL-10/TGF-β, cDC1 density, and TLS maturation. Spatial proximity alone should be supplemented by evidence of uptake.	Evaluate death-inducing therapy with selective efferocytosis-receptor modulation or macrophage reprogramming in preclinical models; clinical translation should be limited to agents with an established safety framework.	Broad inhibition of apoptotic-cell clearance can cause unresolved inflammation and tissue injury. TAM receptors are not interchangeable, and receptor expression alone does not prove active efferocytosis.	Translational maturity: early preclinical/clinical exploration. AXL inhibition with bemcentinib or anti-AXL antibody has preclinical RCC support (95); cabiralizumab (anti-CSF1R) entered an early-phase combination study including advanced RCC (NCT03502330). MERTK/TIM-4-directed strategies are not established in RCC.
Vascular–stromal immune-excluded niche	Dysfunctional vessels, hypoxia, and a collagen-rich CAF/ECM compartment separate PCD-positive tumor regions from cDC1 and CD8^+^ T cells. The limiting step is physical and functional access to dying-cell material.	Assess vessel architecture and CD31, perfusion or hypoxia markers, α-SMA/CAF states, collagen density and orientation, T-cell penetration depth, and distances from PCD-positive tumor cells to cDC1/CD8^+^ T cells and TLS.	Use clinically established ICI–VEGFR-TKI approaches where indicated; evaluate additional matrix-directed intervention only when a dominant stromal barrier is demonstrated and prospectively measured.	Antiangiogenic treatment is dose- and time-dependent and may either normalize or excessively prune vessels. CAF programs are heterogeneous, so indiscriminate stromal depletion may be counterproductive.	Translational maturity: clinically established for ICI–VEGFR-TKI combinations, but the proposed PCD-niche mechanism is not prospectively isolated. Spatially selected matrix-directed therapy remains investigational (12–29, 68, 69, 80, 81).
Inflammatory PCD with myeloid diversion	Gasdermin- or MLKL-associated lytic signaling is present, yet DAMP/IL-1 activity is accompanied by MDSC/Treg accumulation, abnormal vasculature, and stromal repair rather than productive T-cell priming.	Combine cleaved GSDMD/GSDME or pMLKL with mature IL-1β/IL-18, cDC1 activation, MDSC/Treg density, TAM state, chemokines, vessel remodeling, and CAF activation in the same spatial region.	Evaluate controlled inflammatory PCD together with a myeloid- or IL-1-axis intervention only in preclinical or biomarker-enriched settings where lytic execution is verified and immune routing is suppressive.	Both inadequate inflammation and excessive inflammation are plausible. Blocking IL-1 may reduce myeloid suppression but could also blunt useful innate activation; timing and tissue toxicity are central.	Translational maturity: conceptual/preclinical. Direct ccRCC evidence linking gasdermin or pMLKL execution to a suppressive myeloid routing phenotype and treatment response remains sparse (41–59).
TLS-positive niche with metabolically impaired effector T cells	TLS and antigen presentation are present, but CD8^+^ T cells show high lipid uptake, oxidative damage, terminal exhaustion, or ferroptotic vulnerability. The bottleneck lies in effector-cell fitness after priming.	Characterize TLS maturity and B/T-cell organization, TCR clonality, cDC1 proximity, and CD8^+^ T-cell IFN-γ/TNF, exhaustion markers, CD36, lipid ROS, and viability. Distinguish reversible dysfunction from terminal loss.	Evaluate ICI combinations with T-cell-directed metabolic or lipid-homeostasis support designed to preserve effector function without protecting tumor cells from ferroptosis.	A metabolic target may have opposite effects in tumor and immune cells. Apparent TLS positivity is not sufficient if the structure is immature or spatially disconnected from tumor antigen.	Translational maturity: biomarker/observational evidence is increasingly strong for TLS and spatial immune states, whereas T-cell-directed metabolic rescue remains exploratory (27–29, 38–40, 80, 81).

These archetypes are proposed research strata rather than treatment categories. The final column explicitly distinguishes clinically established therapy, early clinical exploration, preclinical evidence, and conceptual hypotheses. Prospective validation should define assay thresholds, reproducibility, temporal stability, cell-type selectivity, and toxicity.

CAF, cancer-associated fibroblast; cDC1, type 1 conventional dendritic cell; DAMP, damage-associated molecular pattern; ECM, extracellular matrix; ICI, immune checkpoint inhibitor; ICD, immunogenic cell death; MDSC, myeloid-derived suppressor cell; MDA, malondialdehyde; PCD, programmed cell death; TAM, tumor-associated macrophage; TCR, T-cell receptor; TLS, tertiary lymphoid structure; Treg, regulatory T cell; VEGFR-TKI, vascular endothelial growth factor receptor tyrosine kinase inhibitor.

### 8.3 A continuous validation chain from cellular source to treatment response

Mechanistic validation should proceed through five linked stages. First, establish the cellular source of death by co-localizing lineage markers with PCD execution markers in malignant cells, CD8^+^ T cells, TAMs, cDC1, or other stromal and immune populations. Second, show that the relevant program has reached execution rather than merely a primed or stressed state. Ferroptosis requires lipid peroxidation, iron dependence, and functional rescue with a selective intervention; pyroptosis requires GSDMD or GSDME cleavage, generation of the pore-forming N-terminal fragment, and pore-dependent membrane permeability; necroptosis requires MLKL phosphorylation, oligomerization or membrane localization, ideally with RIPK3-MLKL pathway intervention; and apoptosis requires caspase/PARP cleavage, pathway activation, and compatible morphology. Third, identify the cells that receive and process death-derived signals by quantifying distances, neighborhoods, and direct contacts with cDC1, CD8^+^ T cells, TAMs, TLS, vessels, and CAF/ECM. Fourth, define immune consequence through dendritic-cell maturation, antigen cross-presentation, T-cell receptor clonal expansion, IFN-γ/TNF activity, IL-10/TGF-β, and myeloid suppression. Fifth, link execution, spatial, and functional measurements to treatment response and test key causal steps through genetic blockade, pharmacological inhibition, or rescue. Together, these stages form a continuous chain from cellular source to execution, spatial reception, immune outcome, and clinical response (17–58, 60, 63–67, 70–74, 82, 83).

Sampling should prioritize pretreatment, early on-treatment, and progression material when feasible, with matched blood, imaging, and clinical outcomes. In practice, intralesional heterogeneity, interlesional divergence, needle-biopsy sampling bias, limited tissue volume, and the morbidity of serial metastatic biopsies constrain this ideal design. Multi-region sampling at surgery, image-guided biopsy of biologically distinct lesions, and paired radiomic or circulating correlates may partially mitigate these limitations. Spatial transcriptomics can discover cellular neighborhoods and ligand-receptor networks; spatial proteomics and multiplex immunofluorescence can validate execution proteins and cellular source; and organoids or *in vivo* models are required for causal perturbation. No single platform can replace the complete source → execution → spatial reception → immune function → treatment-response chain (17–29, 79, 92–94).

## 9 Discussion: limitations and priorities for future research

Current ccRCC research on PCD/RCD has four major limitations. First, many studies rely on public transcriptomic datasets, pathway scores, and prognostic models without execution-level evidence such as protein cleavage, lipid peroxidation, execution-protein phosphorylation/membrane localization, or pathway-specific rescue. Second, malignant, immune, and stromal compartments are often analyzed together, obscuring cell-type-specific and sometimes opposing effects. Third, most studies use pretreatment cross-sectional specimens and cannot resolve treatment-induced remodeling. Fourth, emerging modalities such as cuproptosis and disulfidptosis are increasingly represented by signatures, whereas direct patient-tissue execution and spatial immune consequences remain substantially less mature than their transcriptomic literature (30, 59, 60).

Several questions should be prioritized. Which ferroptotic, pyroptotic, and necroptotic events can be demonstrated directly in RCC patient tissue with clear cellular assignment, spatial localization, and functional support? How can tumor-cell PCD be distinguished in the same region from death occurring in CD8^+^ T cells, natural killer cells, TAMs, endothelial cells, or other stromal populations, and what is the net effect on treatment response? Are efferocytosis-active TAMs an antecedent driver of primary resistance or a secondary state that emerges after treatment failure and progression? Can mature TLS convert treatment-released tumor antigens into newly generated and durable B- and T-cell responses? How does HIF-2α inhibition reshape tumor-cell ferroptosis thresholds, vascular function, and myeloid ecology? Finally, how can high-dimensional spatial multi-omics signals be reduced to stable, reproducible biomarker panels suitable for multicenter validation and clinical use (17–29, 34–39, 41–60)?

Most mechanistic evidence currently derives from ccRCC. Papillary RCC, chromophobe RCC, collecting duct carcinoma, renal medullary carcinoma, and other non-clear-cell subtypes differ substantially in cellular origin, molecular drivers, metabolic dependencies, vascular features, and immune ecology. A PCD-associated immune-niche framework developed in ccRCC should therefore not be transferred to these subtypes without validation. Future work should construct subtype-specific spatial maps of death execution and immune routing and determine whether these niches predict response to ICIs, VEGFR-TKIs, mTOR inhibitors, or other mechanism-based therapies (68, 96–99). Recent pathology and genomic studies of low-grade oncocytic renal tumors and FH-deficient hereditary renal cancer further illustrate the biological heterogeneity of renal neoplasia, although they do not directly establish PCD-associated immune niches (100, 101).

## 10 Conclusions

PCD/RCD forms a critical interface between metabolic vulnerabilities in ccRCC and immune selection pressure, but clinical significance cannot be inferred from a pathway label or transcript signature. Tissue architecture first determines whether dying cells and their released signals are accessible; execution determines what material is generated; and heterogeneous recipient populations, including cDC1, macrophage states, NK cells, CD8^+^ T cells, and TLS-associated cells shape the subsequent immune response. cDC1-associated cross-presentation and TAM-associated efferocytosis should therefore be interpreted as representative axes within a continuum rather than binary outcomes. Future studies should move beyond bulk PCD scores toward a continuous evidence chain linking cellular source, death execution, spatial neighborhood, recipient-cell state, and treatment response. This framework may ultimately support mechanism-guided patient stratification, but most niche-directed interventions still require prospective validation.
